# An Initial Attack of Urinary Stone Disease Is Associated with an Increased Risk of Developing New-Onset Irritable Bowel Syndrome: Nationwide Population-Based Study

**DOI:** 10.1371/journal.pone.0157701

**Published:** 2016-06-23

**Authors:** Wei-Yuan Lei, Chih-Yu Chang, Jr-Hau Wu, Fei-Hung Lin, Cheng Hsu Chen, Chin-Fu Chang, Yan-Ren Lin, Han-Ping Wu

**Affiliations:** 1 Department of Emergency Medicine, Changhua Christian Hospital, Changhua, Taiwan; 2 Department of Biological Science and Technology of Biochemical Engineering, National Chiao Tung University, Hsinchu, Taiwan; 3 School of Medicine, Chung Shan Medical University, Taichung, Taiwan; 4 School of Medicine, Kaohsiung Medical University, Kaohsiung, Taiwan; 5 Division of Pediatric General Medicine, Department of Pediatrics, Chang Gung Memorial Hospital at Linko, Kweishan, Taoyuan, Taiwan; 6 College of Medicine, Chang Gung University, Taoyuan, Taiwan; Université catholique de Louvain, BELGIUM

## Abstract

**Background:**

The neurotransmitter pathways in irritable bowel syndrome (IBS) and urinary stone attacks are both related to serotonin, and each disease may be influenced by viscero-visceral hyperalgesia. However, the relationship between urinary tract stone disease and IBS has never been addressed. We aimed to investigate the risk of suffering new-onset IBS after an initial urinary stone attack using a nationwide database.

**Methods:**

A study group enrolled a total of 13,254 patients who were diagnosed with an initial urinary stone attack; a comparison group recruited 39,762 matched non-urinary stone participants during 2003 and 2007. We followed each patient for 3 years to determine new-onset IBS. We also used Cox proportional hazards models to analyze the risk of IBS between the study and comparison groups after modified by demographics, residence, patient characteristics and personal histories.

**Results:**

The occurrence rates of IBS were 3.3% (n = 440) and 2.6% (n = 1,034) respectively in the study and comparison groups. A covariate-adjusted hazard ratio (HR) of IBS in the study group that was 1.28 times greater (HR = 1.29, 95% CI, 1.15–1.44) than that in the comparison group was showed in the stratified Cox proportional analysis. The adjusted HRs of IBS did not decrease after considering demographics and past histories. The majority of IBS (30.5%) occurred within the first 6 months after the stone attack.

**Conclusion:**

Patients with an initial urinary stone attack are at increased risk of developing new-onset IBS. The HRs of IBS did not decrease even after adjusting for patient demographics and past histories. Most importantly, 30.5% of IBS occurred within the first 6 months after the urinary stone attack.

## Introduction

Irritable bowel syndrome (IBS) has a high incidence worldwide and affects 10%-15% of population in Western countries. IBS is characterized by chronic, recurrent gastrointestinal upset followed by bowel dysfunction.[1–3] Additionally, it has a huge impact on healthcare costs and quality of life. In the United States, the annual cost of IBS treatment has been calculated to be $20 billion in indirect costs and range between $1.7 billion and $10 billion in direct medical costs.[4,5] In spite of its high prevalence rate, the exact pathophysiology of IBS is not yet clarified. Various mechanisms, such as gastrointestinal dysmotility[6,7], visceral hypersensitivity[8,9], intestinal mucosa activation[10–14], and increased intestinal permeability[8,9], have been implicated in the pathophysiology of IBS. Among these, visceral hypersensitivity is considered to be the major cause of IBS.[15,16] Previous analyses of visceral hypersensitivity have identified serotonin (5-hydroxytryptophan, 5-HT) as an crucial neurotransmitter in the pathogenesis of IBS via purinergic mechanosensory transduction.[17–21]

Urinary stone disease is relatively common, with a lifetime risk of approximately 6% in women and 12% in men.[22] Urinary stones in the ureter or urinary bladder can induce distension of tubes or sacs, leading to the release of adenosine triphosphate (ATP) from the epithelium lining the tube or sac, which triggers sensory nerves to convey information to the central nerve system (CNS), causing visceral pain. Similar to the neurotransmitter pathway of IBS, patients with urinary stone attacks also suffer visceral pain via purinergic mechanosensory transduction, and serotonin can be released from enterochromaffin cells.[21,23,24] In addition, certain previous studies reported that viscero-visceral hyperalgesia between two internal organs in the same patient might enhance pain symptoms, potentially via sensitization of viscero-viscero-somatic convergent neurons.[25–28] One of these studies further investigated the interactions between dysmenorrhea and urinary stone disease and between IBS and dysmenorrhea; they noted that treating one disease might reduce the symptoms of the other disease.[25]

Because of the similar neurotransmitter pathways linked to IBS and urinary stone attacks and the possibility of viscero-visceral hyperalgesia, we suspected that IBS could potentially be induced by urinary stone disease. However, the relationship between IBS and urinary stone disease has never been discussed. In this study, we analyze the relationship between the diagnosis of an initial urinary stone attack and the risk of new-onset IBS during a three-year follow-up period by using a nationwide population database.

## Materials and Methods

### Database

In this study, the data were randomly enrolled from the Longitudinal Health Insurance Database (LHID), which was developed by the National Health Insurance (NHI) program. As described previously [29], this program enrolled almost 99% of Taiwan’s population and obtained original data from one million people.

### Ethics statement

Because the data that we used is de-identified secondary data; therefore, our study was exempt from a full review by the Institutional Review Board (IRB). (IRB of Changhua Christian Hospital, permission code: 150216). This manuscript has also followed the Strengthening the Reporting of Observational Studies in Epidemiology (STROBE) guidelines.

### Study setting and population

The data of this retrospective cohort study were collected from the LHID during the period from January 1, 2003 to December 31, 2007. The study group was defined as patients who suffered an initial urinary stone attack. The comparison group was defined as patients who never suffer any urinary stone attacks. In this analysis, the study patients (with an initial urinary stone attack) and the comparison patients (without urinary stones) were followed for 3 years. The chance of suffering a new-onset episode of IBS was analyzed for the two groups during the 3-year follow-up period.

### Inclusion criteria

#### Definition of patients with an initial urinary stone attack

Patients who were diagnosed principally (the major reason for coming to the hospital) as urinary stone attack by an emergency or outpatient department using the International Classification of Diseases, 9th Revision, Clinical Modification codes (using ICD-9-CM; codes 592, 592.1 and 592.9) were enrolled in the study.

#### Definition of patients with irritable bowel syndrome

We defined IBS patients as those who were diagnosed according to the criteria of the International Classification of Diseases, 9th Revision, Clinical Modification (using ICD-9-CM; code 564.1) by an emergency or outpatient department.

### Exclusion criteria

Patients with any of the following characteristics were excluded from this study:

1. Age <18 years.
2. Diagnosis of any form of urinary tract stone (including acute/chronic attack or no prior attack) or IBS before the study period.
3. Inability to be followed up during the study period (i.e., death or incomplete medical records).
4. Urinary stone disease was the co-diagnosis (not the major reason for the hospital visit).

### Quality control

Overtreatment and ICD-9 over-coding were not permitted in the NHI program; these actions could result in fines. Specialists routinely and randomly inspected the treatments, diagnosis, and medications for each patient.

### Study protocol

Our study group included 13,254 patients who suffered a first urinary stone attack. The comparison group was chosen from the remaining NHI beneficiaries registered in the LHID. 39,762 comparison patients (three comparison patients for each urinary stone patient) who were matched to the study group by age, gender, and number of hospital visits and years of index healthcare use were randomly enrolled. This study included a total of 53,016 patients.

### Data analysis

The SAS program (SAS Institute Inc., Cary, NC, USA) was used to select the study and comparison groups. We followed each patient (n = 53,016) for three years to identify those who experienced new-onset IBS. The independent variables, including personal history at baseline, patient characteristics, and demographics, are reported as percentages or the mean ± standard deviation (SD).

We used the X^2^ test to analyze the differences between the study and comparison groups for demographics, including socioeconomic level (>$1,000 USD, $601–1,000 USD or <$600 USD, monthly income of the patient and guardian), the degree of urbanization in the patient’s city of residence, the location of the patient’s residence (eastern and western Taiwan; western Taiwan was further divided into northern, central and southern Taiwan), and personal disease history (diabetes, hypertension, liver cirrhosis, renal failure, stroke, osteoporosis and fibromyalgia). The degree of urbanization, which was classified by population and certain development-related conditions, (Level 1 urbanization: more than 1,250,000 people; level 2: 1,250,000~500,000 people; levels 3: 500,000~150,000 people; level 4: fewer than 150,000 people).[30] Moreover, the crude hazard ratio (HR) was calculated by creating age-stratified Cox proportional hazards models, which were used in the study and comparison groups to analyze the risk of experiencing new-onset IBS. Furthermore, the HR was analyzed after adjusting for demographics (mode 1), personal disease history (mode 2), and all variables (mode 3).

Moreover, variables that were related or unrelated to the occurrence of IBS among the study and comparison patients were analyzed using the X^2^ test. These variables included demographics and personal disease history. Furthermore, multiple logistic regression analysis was respectively used to analyze the more important factors that associated with new-onset IBS for all patients, study patients and comparison patients.

We also used the log-rank test and the Kaplan-Meier method to estimate 3-year IBS-free survival rates for the study and comparison groups. Finally, among the patients with an initial urinary stone attack, the amount of time before the onset of IBS was recorded and further divided into 6 periods (<6, 6–12, 13–18, 19–24, 25–30, and 31–36 months). P<0.05 was considered to indicate statistical significance.

## Results

### 1. Demographics and personal histories collected from patients with an initial urinary stone attack

The characteristics of patients with an initial urinary stone attack (n = 13,254) and control patients (without any urinary stone history; n = 39,762) are showed in Table 1. Males comprised the preponderance in both groups. Most of the initial urinary stone attacks arose in the age groups of 30 to 39 years (24.1%) and 40 to 49 years (25.4%). Compared with the control patients, the economic and urbanization levels were significantly lower in those with urinary stones. Additionally, patients with urinary stones had a higher prevalence of diseases, including hypertension, renal failure, osteoporosis and fibromyalgia (for all of the above findings, p<0.05).

**Table 1:** Characteristics and personal histories between patients with urinary stone attack and comparison patients.

	Patients with Urinary Stone Attack (n = 13,254)		Comparison Patients (n = 39,762)		
	No.	%	No.	%	*p*
**Gender**					1.000
Male	9,251	69.8	27,753	69.8	
**Mean age (y/o) (mean±SD)**	45.3±14.3		45.1±14.6		0.210
**Age group (y/o)**					1.000
≤30	2,052	15.5	6,156	15.5	
30–39	3,197	24.1	9,591	24.1	
40–49	3,363	25.4	10,089	25.4	
50–59	2,515	19.0	7,545	19.0	
60–69	1,310	9.9	3,930	9.9	
70–79	657	5.0	1,971	5.0	
≥80	133	1.1	465	1.2	
Economic level (monthly income) (USD$)*					<0.001
<600	3,547	26.8	10,419	26.2	
601–1,000	6,609	49.9	18,816	47.3	
>1,000	3,098	23.4	10,527	26.5	
Urbanization*					<0.001
1 (most)	3,584	27.1	11,003	29.0	
2	1,539	11.7	4,741	12.5	
3	3,920	29.4	10,708	28.3	
4	4,211	31.8	11,815	29.7	
Geographic regions of Taiwan*					<0.001
Northern	6,927	52.3	20,502	51.6	
Central	3,055	23.0	8,501	21.4	
Southern	2,794	21.1	9,580	24.1	
Eastern	478	3.6	1,179	3.0	
**Personal history**					
Diabetes mellitus	950	7.2	2,762	6.9	0.387
Hypertension*	1,754	13.2	4,848	12.2	0.002
Renal failure*	342	2.6	861	2.2	0.006
Liver cirrhosis	1,065	8.0	3,052	7.7	0.183
Stroke	81	0.6	306	0.8	0.069
Osteoporosis*	2,292	17.3	6,575	16.5	0.044
Fibromyalgia*	4,503	34.0	11,742	29.5	<0.001

*Significant differences, *p*<0.05.

### 2. Irritable bowel syndrome probability based on crude HR

We found that the risk of suffering new-onset IBS was significantly higher in the study patients than in the comparison patients during the 3-year follow-up period. We found that 3.3% (n = 440) of patients experienced IBS after the occurrence of an initial urinary stone attack, whereas the percentage of IBS was only 2.6% (n = 1,034) in the comparison patients. The study patients had a crude HR 1.29 times greater than that of the comparison patients (95% CI, 1.15–1.44; p<0.001) (Table 2), which is showed in the stratified Cox proportional hazards analysis. Furthermore, adjusting for likely influence factors, including patient geographic region, monthly income (mode 1), personal history at baseline (mode 2) and both modes 1 and 2 (mode 3), did not affect the HRs of suffering IBS, which all remained higher in patients with an initial stone attack compared with controls (for all of the above findings, p<0.05) (Table 3).

**Table 2:** Crude HRs for new-onset irritable bowel syndrome among patients with urinary stone attack and comparison patients.

New-Onset IBS	Total Sample (n = 53,016)		Patients with Urinary Stone Attack (n = 13,254)		Comparison Patients (n = 39,762)	
3-year follow-up	No.	%	No.	%	No.	%
Yes	1,474	2.8	440	3.3	1,034	2.6
No	51,542	97.2	12,814	96.7	38,728	97.4
**Crude HR (95% CI)**	-		1.29* (1.15–1.44)		1.00	

*p<0.0001. HR, hazard ratio; IBS, irritable bowel syndrome.

**Table 3:** Adjusted-effect estimates for urinary stone attack.

	Occurrence of new-onset irritable bowel syndrome					
	Mode 1		Mode 2		Mode 3	
Variables	HR	95% CI	HR	95% CI	HR	95% CI
**Groups**						
Patients with urinary stone	1.29	1.15–1.44	1.26	1.13–1.41	1.28	1.13–1.42
Control*	1.00	-	1.00	-	1.00	-
**Geographic regions**						
Northern*	1.00	-	-	-	1.00	-
Central	0.99	0.86–1.14	-	-	0.98	0.85–1.13
South	1.01	0.88–1.15	-	-	0.99	0.87–1.14
Eastern	0.63	0.44–0.91	-	-	0.62	0.43–0.89
**Economic level (monthly income, USD$)**						
>1,000*	1.00	-	-	-	1.00	-
600–1,000	0.92	0.81–1.04	-	-	0.92	0.81–1.05
<600	0.85	0.74–0.99	-	-	0.87	0.75–1.01
**Urbanization**						
1*	1.00	-	-	-	1.00	-
2	1.10	0.92–1.31	-	-	1.09	0.91–1.30
3	0.99	0.86–1.14	-	-	0.98	0.85–1.13
4	1.11	0.95–1.28	-	-	1.11	0.96–1.28
**Personal history**						
Diabetes	-	-	1.08	0.88–1.31	1.08	0.88–1.31
Hypertension	-	-	1.12	0.95–1.32	1.12	0.95–1.32
Renal failure	-	-	1.28	0.94–1.74	1.27	0.93–1.73
Liver cirrhosis	-	-	1.62	1.37–1.93	1.61	1.36–1.91
Stroke	-	-	1.84	1.19–2.85	1.86	1.20–2.88
Osteoporosis	-	-	1.36	0.18–1.57	1.36	1.18–1.57
Fibromyalgia	-	-	1.33	1.19–1.48	1.33	1.19–1.48

*Reference group. Mode 1: Adjusted by demographics (i.e., economic level of family, degree of urbanization and geographical location). Mode 2: Adjusted by personal disease histories (i.e., diabete, hypertension, renal failure, liver cirrhosis, stroke, osteoporosis and fibromyalgia). Mode 3: Adjusted by demographics and personal disease histories.

### 3. Clinical features associated with the occurrence of IBS in patients with an initial urinary stone attack (n = 440)

The clinical features of patients in both the study and comparison groups who suffered new-onset IBS are shown in Table 4. We found that IBS was more predominant in the age group of 40 to 49 years and in those with a history of osteoporosis and fibromyalgia in both the study and comparison groups (all p<0.05). Furthermore, diabetes was significantly associated with IBS only in the study patients (p<0.05). The results of multiple logistic regression analysis of suffering new-onset IBS for all patients, study patients and comparison patients are shown in Table 5.

**Table 4:** Clinical features associated with new-onset irritable bowel syndrome in patients with urinary stone attack.

	Patients with Urinary Stone Attack (n = 13,254)		Comparison Patients (n = 39,762)	
	New-Onset IBS (n = 440) No. (%)	*p*	New-Onset IBS (n = 1,034) No. (%)	*p*
**Gender**		0.056		0.001
Male	289 (65.7)		674 (68.2)	
**Mean age**	47.9±14.8		49.1±15.6	
Age group (y/o)*		0.003		<0.001
≤30	52 (11.8)		122 (11.8)	
30–39	94 (21.4)		195 (18.9)	
40–49	107 (24.3)		231 (22.3)	
50–59	94 (21.4)		231 (22.3)	
60–69	52 (11.8)		145 (14.0)	
70–79	37 (8.4)		86 (8.3)	
≥80	4 (0.9)		24 (2.3)	
**Economic level (monthly income) (USD$)**		0.494		0.645
<600	107 (24.3)		259 (25.0)	
600–1,000	228 (51.8)		492 (47.6)	
>1,000	105 (23.9)		283 (27.4)	
**Geography regions of Taiwan**		0.266		0.241
Northern	229 (52.2)		530 (51.3)	
Central	96 (21.9)		236 (22.8)	
Southern	103 (23.5)		247 (23.9)	
Eastern	11 (2.5)		21 (2.0)	
**Urbanization**		0.403		0.171
1 (most)	114 (25.9)		294 (28.4)	
2	62 (14.1)		133 (12.9)	
3	124 (28.2)		271 (26.2)	
4	140 (31.8)		336 (32.5)	
**Personal history**				
Diabetes mellitus*	46 (10.5)	0.009	70 (6.8)	0.895
Hypertension	51 (11.6)	0.352	164 (15.9)	<0.001
Renal failure	16 (3.6)	0.166	27 (2.6)	0.330
Liver cirrhosis	35 (8.0)	0.991	131 (12.7)	<0.001
Stroke	6 (1.4)	0.052	15 (1.5)	0.018
Osteoporosis*	103 (23.4)	<0.001	219 (21.2)	<0.001
Fibromyalgia*	195(44.3)	<0.001	388(37.5)	<0.001

*Significant features associated with irritable bowel syndrome in study group, *p*<0.05. IBS, irritable bowel syndrome.

**Table 5:** Multiple logistic regression analysis of suffering new-onset IBS for all patients, study patients and comparison patients.

	All patients (n = 53,016)		Patients with Urinary Stone Attack (n = 13,254)		Comparison Patients (n = 39,762)	
Variables	OR	95% C.I.	OR	95% C.I.	OR	95% C.I.
**Gender**						
Female*	1.00	-	1.00	-	1.00	-
Male	0.87	0.78–0.98	0.91	0.74–1.12	0.86	0.75–0.98
**Age group (y/o)**						
≤30*	1.00	-	1.00	-	1.00	-
30–39	1.01	0.83–1.22	1.13	0.80–1.60	0.96	0.76–1.21
40–49	1.06	0.88–1.28	1.16	0.83–1.64	1.03	0.82–1.29
50–59	1.29	1.06–1.57	1.30	0.90–1.86	1.30	1.03–1.64
60–69	1.48	1.19–1.85	1.38	0.91–2.10	1.53	1.18–1.99
70–79	1.85	1.44–2.38	1.97	1.25–3.12	1.81	1.34–2.45
≥80	1.78	1.17–2.71	0.95	0.33–2.69	2.13	1.34–3.39
**Economic level (monthly income, USD$)**						
<600*	1.00	-	1.00	-	1.00	-
601–1,000	1.05	0.93–1.20	1.11	0.87–1.40	1.02	0.88–1.20
>1,000	1.13	0.97–1.31	1.12	0.85–1.49	1.14	0.95–1.35
**Geographic regions**						
Northern*	1.00	-	1.00	-	1.00	-
Central	0.98	0.84–1.13	0.88	0.67–1.15	1.02	0.86–1.22
Southern	0.98	0.86–1.12	1.08	0.85–1.39	0.96	0.82–1.12
Eastern	0.62	0.43–0.89	0.58	0.30–1.12	0.64	0.41–0.99
**Urbanization**						
1 (most) *	1.00	-	1.00	-	1.00	-
2	1.09	0.90–1.30	1.33	0.95–1.85	0.99	0.80–1.25
3	0.98	0.85–1.14	1.06	0.81–1.39	0.95	0.80–1.13
4	1.11	0.96–1.29	1.11	0.84–1.46	1.10	0.92–1.32
**Diabetes mellitus**						
Yes	1.08	0.88–1.33	1.46	1.04–2.04	0.92	0.71–1.19
No*	1.00	-	1.00	-	1.00	-
**Hypertension**						
Yes	1.13	0.95–1.34	0.82	0.59–1.15	1.26	1.04–1.54
No*	1.00	-	1.00	-	1.00	-
**Renal failure**						
Yes	1.29	0.94–1.76	1.41	0.84–2.38	1.21	0.81–1.80
No*	1.00	-	1.00	-	1.00	-
**Liver cirrhosis**						
Yes	1.62	1.36–1.93	0.99	0.69–1.43	1.93	1.58–2.35
No*	1.00	-	1.00	-	1.00	-
**Stroke**						
Yes	1.87	1.19–2.94	2.19	0.93–5.15	1.79	1.05–3.05
No*	1.00	-	1.00	-	1.00	-
**Osteoporosis**						
Yes	1.37	1.19–1.59	1.34	1.03–1.74	1.37	1.16–1.63
No*	1.00	-	1.00	-	1.00	-
**Fibromyalgia**						
Yes	1.35	1.21–1.50	1.46	1.20–1.78	1.29	1.13–1.47
No*	1.00	-	1.00	-	1.00	-

*Reference group. OR, Adjusted odds ratio; C.I., confidence interval.

### 4. IBS-free survival curves for patients

**Fig 1:**
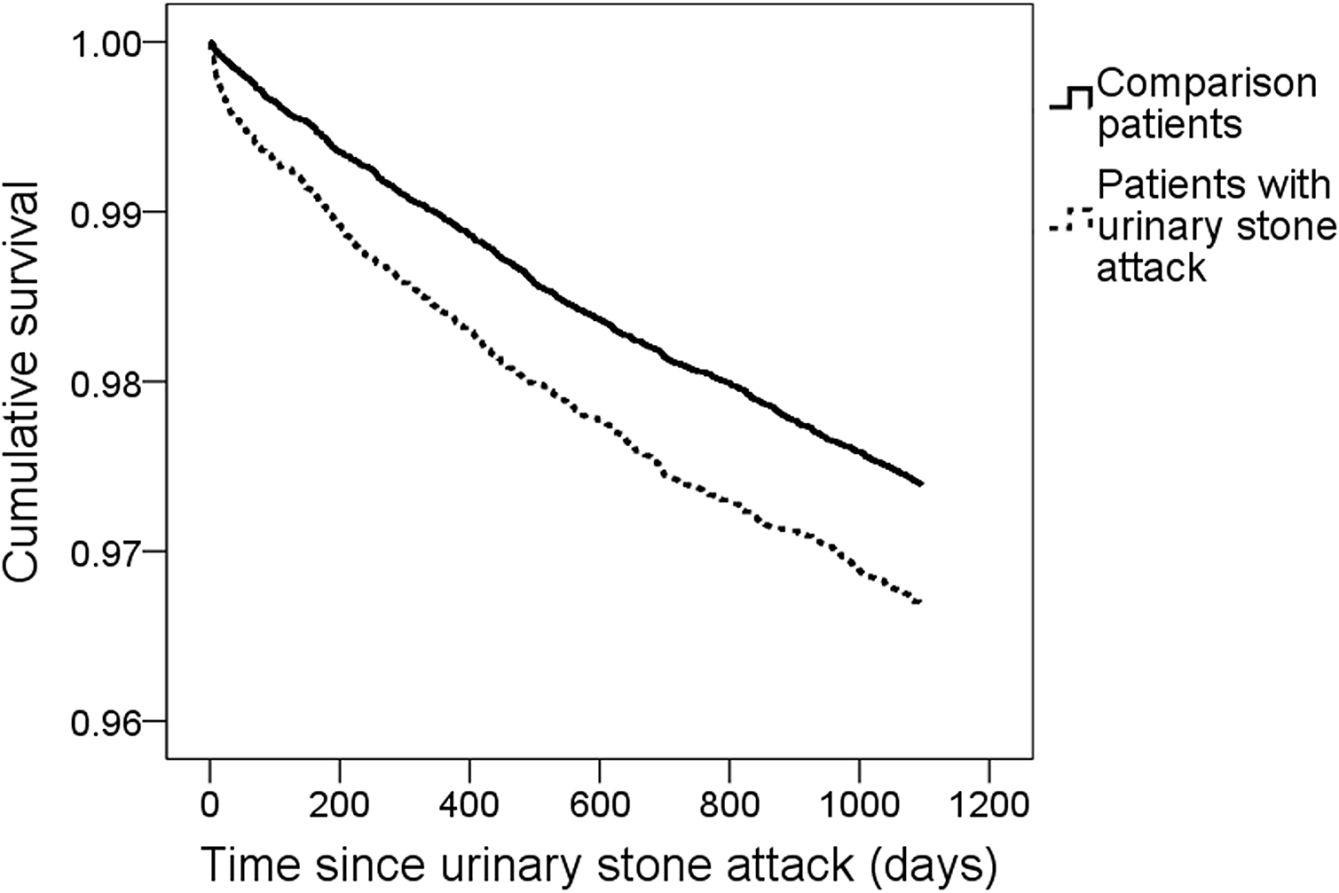
Time-related factors associated with new-onset irritable bowel syndrome occurrence. Irritable bowel syndrome-free survival curves for patients with urinary stone attack and comparison patients during the 3-year follow-up period (*p* = 0.001).

The IBS-free survival curves of study and comparison patients generated during the study period are shown in Fig 1. Patients with an initial urinary stone attack had a significantly lower incidence of 3-year IBS-free survival than the comparison patients (p<0.05).

### 5. Time between the initial urinary stone attack and IBS onset

**Fig 2:**
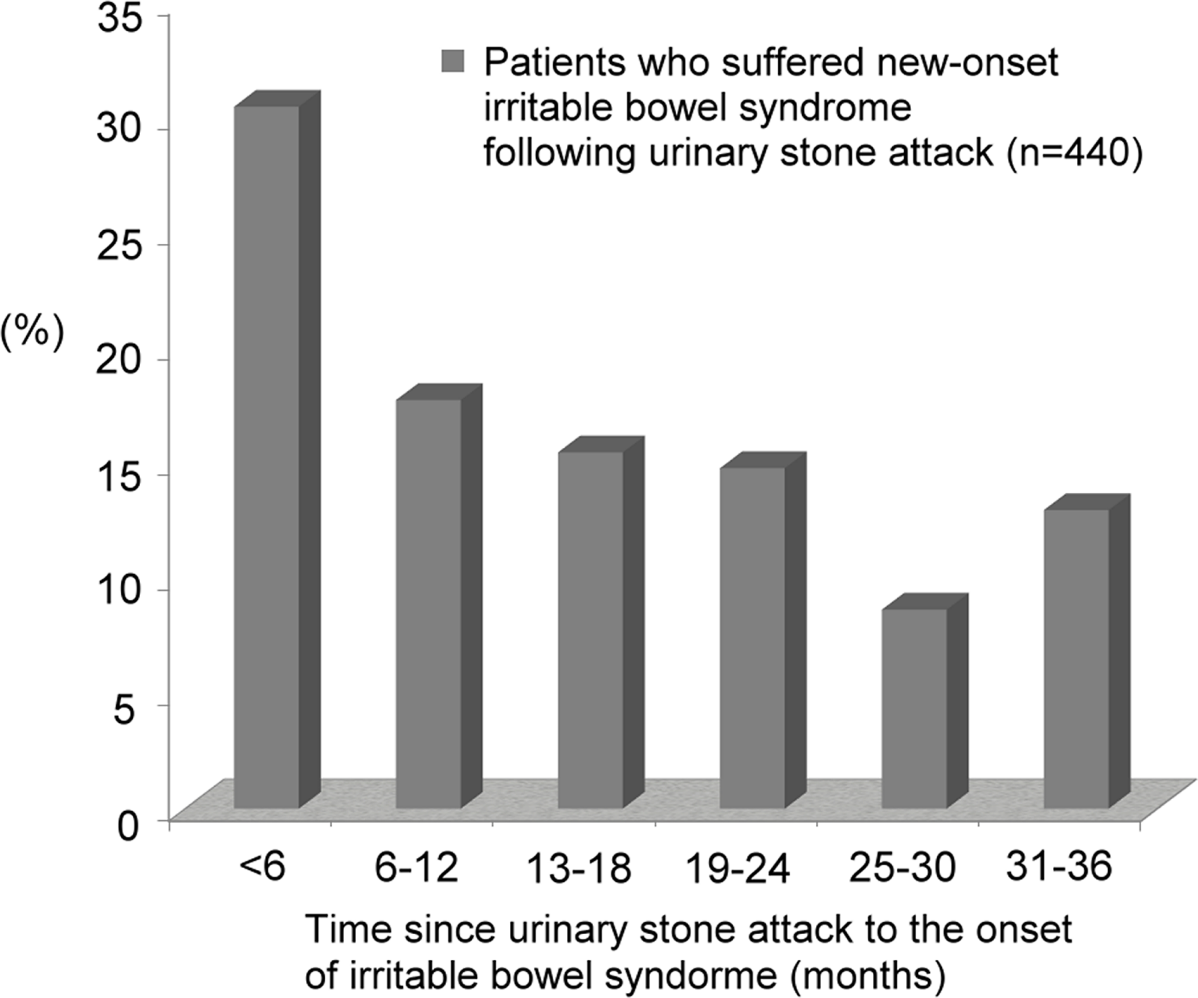
Most irritable bowel syndrome (30.5%) occurred within the first 6 months after the initial urinary stone attack.

The time between experiencing an initial urinary stone attack and new-onset IBS in the study period is shown in Fig 2. Most IBS cases (30.5%) occurred within the first 6 months after the urinary stone attack.

## Discussion

Although the incidence of IBS and its impact on human life is high worldwide[1–3], its pathophysiological mechanisms remain unclear. Several studies have previously reported that the neurotransmitter pathways of IBS and urinary stone attack are similar, with serotonin-related visceral pain or viscero-visceral hyperalgesia [23,25,26,31–34]; therefore, we suspected that urinary stone attack may be a major cause of IBS. One previous study reported that the relative risk of developing IBS was 2.48-fold higher in patients with urinary stone disease than in those without urinary stone disease in a small sample size.[35] However, the relationship between IBS and urinary stone disease has never been well demonstrated in large papulation. In this study, we aimed to analyze the relationship between a diagnosis of first urinary stone attack and the risk of new-onset IBS within a 3-year follow-up period.

Clinically, serotonin plays a key factor in colic pain via purinergic mechanosensory transduction, and it is also the most significant neurotransmitter in the pathogenesis of IBS.[21,31,32] Serotonin is a paracrine signaling molecule found widely throughout the gastrointestinal tract, and it modulates governing functions, such as motility, secretion, sensation, and blood flow.[17–20] It can be released from enterochromaffin cells and the enteric motor system by triggering by chemical signals, especially conducted by luminal distension.[23,34] Serotonin receptors modulate visceral pain and assist in peristalsis, and the emotional component of visceral stimulation seems to be affected through these receptors within the CNS.[36–38] Urinary stones in the ureter or urinary bladder induce distension of tubes or sacs. This leads to the release of ATP from the epithelium lining the tube or sac, which then acts on purinoceptors (P2X3 and P2X2/3) on subepithelial sensory nerves to convey sensory/nociceptive information to the CNS. This neurotransmitter pathway also leads to visceral pain though serotonin release and purinergic mechanosensory transduction.[23,24] Because this neurotransmitter pathway is similar to IBS, we suspected that the IBS may be induced by increased serotonin released from patients with urinary stone attacks. In addition, viscero-visceral hyperalgesia might contribute to IBS in patients with urinary stones. Several previous studies reported that colon-bladder cross-sensitization could induce painful symptoms. Patients with IBS often co-exhibit urinary hypersensitivity.[28,39,40] Moreover, some animal studies on colon-bladder sensitization demonstrated that acute or chronic colon irritation could increase the frequency of bladder contractions and alter micturition reflexes.[27,41] Drug-induced bladder inflammation not only causes bladder hypersensitivity but also induces colon distension.[42,43] Although the mechanisms of viscero-visceral hyperalgesia are not very clear, the most credited hypothesis is that this phenomenon derives from the sensitization of neurons that receiving convergent sensory input from the two affected visceral organs.[25] Sensory afferent information from the bowel and urinary bladder may converge at the level of the dorsal root ganglion (DRG). Noxious stimulus from a directly affected organ to an adjacent non-irritated structure following an inflammatory insult may be transmitted through the hyperexcitability of convergent DRG neurons.[44] Thus, stimulation from urinary stone disease may affect bowel dysmotility via viscero-visceral hyperalgesia and increase the chance of experiencing IBS.

To demonstrate the relationship between urinary stone disease and IBS, we retrospectively reviewed 53,016 (13,254 patients with an initial urinary stone attack and 39,762 matched non-urinary stone participants) cases from LHID in Taiwan and followed each of them for 3 years. According to our results, patients with an initial urinary stone attack were at higher risk for suffering new-onset IBS. Additionally, patients with urinary stones had significantly lower rates of 3-year IBS-free survival compared with control patients. Furthermore, after adjusting for patient demographics (mode 1) and personal histories (mode 2), the HRs of suffering new-onset IBS remained significantly higher in stone attack patients compared with controls. Finally, we found that most IBS occurred within the first 6 months after the initial urinary stone attack. The age distributions were not the same in the groups of patients with an initial urinary stone and those with IBS. We suspect two possible reasons for the slight difference in age distribution: one, sampling bias (this study randomly selected patients from a database); and two, study design (urinary stone was always prior to IBS). Among patients with IBS, a history of osteoporosis and fibromyalgia was more predominant in both the study and control groups. We suspect that malabsorption and inflammation related to IBS are two possible major causes of bone loss and may even induce osteoporosis.[45,46] Otherwise, a disrupted mucosal barrier and altered gut microbiota were observed in patients with IBS, which indicated the relationship between IBS and fibromyalgia may partially reflect disorders in gastrointestinal permeability. [47] In this study, diabetes was associated with IBS only in the study group. We suspect that insulin resistance is associated with defects in renal ammonium production, and acidic urine, which may cause uric acid stones, was more predominant among diabetes patients.[48–51]

According to the results mentioned above, we suggest that physicians consider urinary stone disease before or when IBS is diagnosed. This is especially important for patients previously diagnosed with IBS, as they should be assessed to rule out the likelihood of urinary stone disease. A significant treatment challenge for patients and practitioners remains due to the complex nature of IBS. Antispasmodics, antidepressants, and bulking agents are frequently prescribed for IBS traditionally. Once urinary stone diseases are diagnosed in patients with IBS, passing urinary stones (i.e., extracorporeal shockwave lithotripsy or ureteroscopy [52]) may be considered rather than prescribing the symptomatic treatment for IBS. Otherwise, we suspect that the distension of tubes or sacs (caused by stones) will prolong serotonin release and purinergic mechanosensory transduction; therefore, the symptoms of IBS may not improve.

In conclusion, patients with a first urinary stone attack are at increased risk of developing new-onset IBS. The HRs of suffering IBS did not decrease even after adjusting for patient demographics and prior histories. Most importantly, 30.5% of IBS occurred within the first 6 months after the initial urinary stone attack.

### Limitations

A natural limitation for researchers who analyze data using the LHID database cannot be avoided is that diagnoses made before 1995 were not included since the LHID began in 1995.[53–54] Consequently, we cannot clearly exclude several patients had diagnoses of IBS or urinary stone diseases before 1995. To decrease this limitation, patients who had any medical record of IBS or urinary stone diseases from 1995 to 2003 were excluded. Moreover, ICD-9 over- or miscoding was another limitation. The codes sent to the National Health Database were made by the attending physicians in emergency/outpatient departments. The treating physicians in Taiwan must make and confirm all of the codes by following Taiwan law. Although the Rome criteria (criteria fulfilled for the last 3 months with symptom onset at least 6 months prior to diagnosis) are commonly used to diagnose IBS, physicians may overdiagnose this syndrome because of its various clinical presentations and uncertain pathophysiology. In this study, some patients (30.5%) were diagnosed with IBS within the first 6 months after initial stone attack. We suspect two possible reasons for this result: one, some of these patients might have had symptoms of IBS (but no IBS diagnosis or presentation at the hospital) before the urinary stone attack; and two, physicians might overdiagnose IBS (not following the Rome criteria). The first issue is a natural limitation of a database-based study, but the second issue has been improved by the government-supported NHI program (see the methods section: quality control). The number of fibromyalgia patients might have been overestimated because the particular ICD-9 code, 729.1, could be easily used to code common myalgia or myositis (natural limitation of the ICD-9). Finally, patients with an initial urinary stone attack may have been over-excluded. We excluded patients if their urinary stone diseases were only the co-diagnosis (or not the major reason for their hospital visit) because they may not be suffering a stone attack.
